# Pharmacokinetics of duloxetine self-administered in overdose with quetiapine and other antipsychotic drugs in a Japanese patient admitted to hospital

**DOI:** 10.1186/s40780-021-00189-9

**Published:** 2021-02-03

**Authors:** Koichiro Adachi, Satoru Beppu, Kei Nishiyama, Makiko Shimizu, Hiroshi Yamazaki

**Affiliations:** 1 Laboratory of Drug Metabolism and Pharmacokinetics, Showa Pharmaceutical University, 3-3165 Higashi-tamagawa Gakuen, Machida, Tokyo, 194-8543 Japan; 2 Kyoto Medical Center, Fushimi-ku, Kyoto, 612-8555 Japan

**Keywords:** Flunitrazepam, Pharmacokinetic modeling, Overdose, Trazodone

## Abstract

**Background:**

Combinations of antidepressant duloxetine (at doses of 40–60 mg/day) and other antipsychotics are frequently used in clinical treatment; however, several fatal and nonfatal cases of duloxetine overdose have been documented. We experienced a patient who had taken an overdose of duloxetine (780 mg) in combination with other drugs in a suicide attempt.

**Case presentation:**

The patient was a 37-year-old man (body weight, 64 kg) with a history of gender identity disorder and depression. He intentionally took an overdose of duloxetine in combination with three other antipsychotic drugs (18 mg flunitrazepam, 850 mg quetiapine, and 1100 mg trazodone) and was emergently admitted to Kyoto Medical Center. The patient’s plasma concentration of duloxetine during ambulance transport was 57 ng/ml, and the level was still as high as 126 ng/mL at 32 h after administration. Duloxetine disappeared most slowly from plasma, in contrast to quetiapine, which was the fastest to clear among the four medicines determined in this patient. The observed concentrations of duloxetine in this overdose patient were generally within the 95% confidence intervals of the plasma concentration curves predicted using a physiologically based pharmacokinetic (PBPK) model.

**Conclusion:**

Even if more than 1 h (the generally recommended period) has passed after administration of duloxetine in such overdose cases, gastric lavage and/or administration of activated charcoal may be effective in clinical practice up to 6 h because of the typically slow elimination behavior illustrated by the PBPK model. Pharmacokinetic profiles visualized using PBPK modeling can inform treatment decisions in cases of drug overdose for medicines such as duloxetine in emergency clinical practice.

## Background

Therapeutic drug monitoring is an accepted clinical practice of measuring the levels of specific antipsychotics drugs in blood samples from patients at designated intervals to maintain drug concentrations in the target range [1, 2]. The antidepressant duloxetine is frequently used in combination with other antipsychotics such as quetiapine in the clinical treatment of major depressive disorder. Nevertheless, both fatal and nonfatal cases of duloxetine overdose have been documented [3–8]. The monitoring of plasma concentrations of duloxetine should now be seriously considered in emergency situations and in special populations. However, there are no known reports that provide a comprehensive analysis of blood samples in an overdose setting for duloxetine self-administered with other antipsychotics.

In general, the drug monitoring of steady-state plasma concentrations of individual patients in the clinical setting could be supported by pharmacokinetic models and simulations. Simplified physiologically based pharmacokinetic (PBPK) models can predict drug monitoring results even in emergency rooms. We previously proposed simple PBPK models for direct oral anticoagulant drugs [9, 10], and, in a case of edoxaban overdose, we recently suggested the practical use of such models by paramedical staff in emergency clinical practice [10].

## Case presentation

Here we describe the case of a 37-year-old man (body weight, 64 kg) who intentionally took an overdose of 780 mg duloxetine (usual clinical dose in the range 40–60 mg/day) in combination with antipsychotic drugs flunitrazepam (18 mg: usual range 0.5–2 mg/day), quetiapine (850 mg: usual range 50–600 mg/day), and trazodone (1100 mg: usual range 75–200 mg/day). The patient had a history of gender identity disorder and depression. He had self-administered these medicines in combination as a suicide attempt and was emergently admitted to Kyoto Medical Center. On arrival, the patient’s awareness level as a Glasgow Coma Scale score was eye 2, verbal 2, and motor 4 (E2V2M4), breathing rate was 16 breaths/min, body temperature was 37.1 °C, oxygen saturation was 98% on room air, blood pressure was 124/86 mmHg, and the heart rate was 89 bpm. An electrocardiogram showed normal sinus rhythm with a QTc of 473 ms. The patient was then infused with bicarbonate Ringer’s solution but was not administrated charcoal and did not undergo artificial dialysis. The clinical laboratory results for the patient 1, 32, and 56 h after the self-administered overdose are shown in Table 1. The patient’s awareness level had improved to E4V5M6 and QTc reduced to < 430 ms 35 h after admission to hospital. No abnormalities were found in vital signs at discharge 3 days after admission. We report herein the drug monitoring data for the patient and the results of pharmacokinetic modeling. The findings indicate that predictions using this tool are appropriate for application in an emergency. The ethics committee of Kyoto Medical Center approved this study (18–018).

**Table 1:** Clinical laboratory results in a patient who had taken a single combined oral overdose of duloxetine, flunitrazepam, quetiapine, and trazodone

	Time after administration (h) of oral dose		
	1	32	56
Aspartate aminotransferase (U/L)	15	138	122
Alanine aminotransferase (U/L)	18	27	34
Serum creatinine (mg/dL)	0.66	0.71	0.64
Creatinine clearance (mL/min)	139	129	143

Frozen plasma samples collected from the patient 1 and 32 h after an overdose of a combination of drugs were pharmacokinetically analyzed. The patient gave written informed consent to take part in this study and for its publication. The concentrations of duloxetine, flunitrazepam, quetiapine, and trazodone in the plasma samples were quantified by liquid chromatography using a gradient elution program followed by tandem mass spectrometry systems according to the reported methods [11–15] with slight modifications; the following transitions were used: *m/z* 298 → 154, *m/z* 314 → 268, *m/z* 384 → 253, and *m/z* 372 → 176, for duloxetine, flunitrazepam, quetiapine, and trazodone, respectively. Under the present conditions, duloxetine, flunitrazepam, quetiapine, and trazodone levels in plasma were measurable (≥10 ng/mL) or detectable (≥0.10 ng/mL) each time point. Duloxetine, flunitrazepam, quetiapine, and trazodone were purchased from Fujifilm Wako Pure Chemicals, Osaka, Japan.

**Fig. 1:**
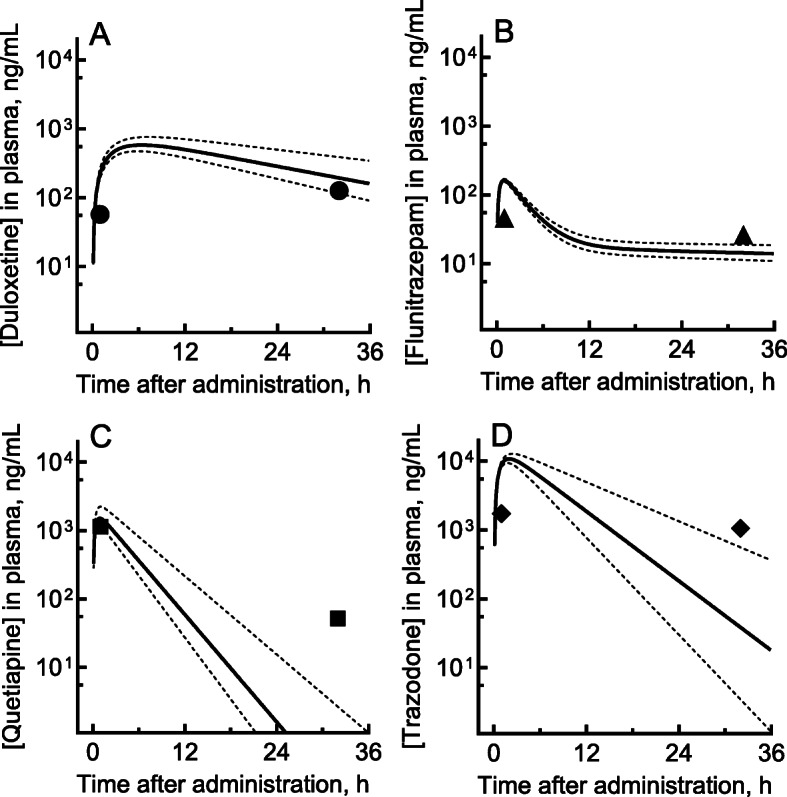
Measured (plots) and estimated (lines) plasma concentrations of duloxetine (**a**), flunitrazepam (**b**), quetiapine (**c**), and trazodone (**d**) in a patient who took a single oral overdose of these drugs. The patient took a single excessive oral dose of duloxetine (780 mg), flunitrazepam (18 mg), quetiapine (850 mg), and trazodone (1100 mg) in combination. The modeled plasma concentration curves after virtual administrations (solid lines) are shown with 95% confidence intervals (broken lines) based on the hepatic intrinsic clearance values shown in Table 2

The patient’s plasma duloxetine concentration during ambulance transport was 57 ng/ml after an oral overdose of 780 mg (Fig. 1), and, 32 h later, the level was still as high as 126 ng/mL. The plasma concentrations at 1 h and 32 h after administration were 46 and 26 ng/mL for flunitrazepam and 1720 and 1060 ng/mL for trazodone, respectively. In contrast, the plasma concentration of quetiapine at 1 h after administration (1140 ng/mL) had rapidly decreased to 52 ng/mL at 32 h. Of the four medicines evaluated in this patient, duloxetine disappeared most slowly from plasma, whereas quetiapine disappeared most quickly.

**Fig. 2:**
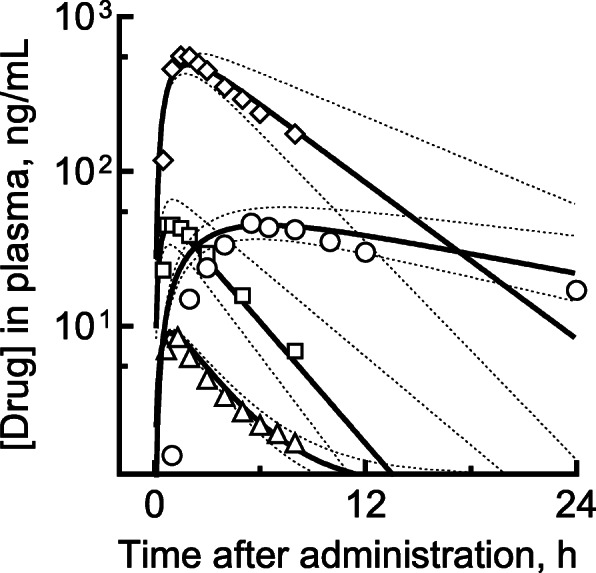
Estimated plasma concentrations (lines) and reported/observed plasma concentrations (plots) of duloxetine (circles), flunitrazepam (triangles), quetiapine (squares), and trazodone (diamonds). Plasma concentration curves after virtual administrations (solid line) are shown with 95% confidence intervals (broken lines) based on the hepatic intrinsic clearance values shown in Table 2. Reported/observed blood levels were taken from the literature: duloxetine (60 mg, [16]), flunitrazepam (1 mg, [17]), quetiapine (25 mg, [18]), and trazodone (50 mg, [19])

Based on the reported human blood concentrations in patients orally treated with the normal therapeutic doses of the four antipsychotic drugs (shown in Fig. 2**)** [16–19], four simple PBPK models consisting of receptor (gut), metabolizing (liver), and central compartments were separately set up as described previously [9, 10, 20, 21]. Rate constants for the transfer of drug from/to the central (first) compartment to/from the peripheral (second) compartment (*k*_12_/*k*_21_) [22] were adopted for flunitrazepam. The plasma unbound fractions (*f*_u,p_), octanol–water partition coefficients (log*P*), blood-to-plasma concentration ratios (*R*_b_), and liver-to-plasma concentration ratios (*K*_p,h_) of the relevant compounds were estimated using in silico tools [9, 23, 24]. The initial values for the fraction absorbed × intestinal availability (*F*_a_·*F*_g_) and hepatic clearance (*CL*_h_) were estimated from the elimination constants in empirical one-compartment models. The absorption rate constant (*k*_a_), volume of the systemic circulation (*V*_1_), and hepatic intrinsic clearance (*CL*_h,int_) values for PBPK models with standard deviations were determined by fitting using nonlinear regression analyses; these final parameters are shown in Table 2 (within 25% of coefficients of variation for *k*_a_, *k*_12_, *k*_21_, *CL*_h,int_, and *V*_1_). The general ratios of *CL*_h_ to the renal clearance (*CL*_r_) were set at 9:1 for the four drugs. The 95% confidence intervals (CIs) were estimated for the fitted intrinsic hepatic clearance values using 100 virtual subjects created using random numbers, as described previously [9, 10]. The resulting system of differential equations was solved to obtain the concentrations of the substrates for the overdosed patient in this study:

$$ \frac{d{X}_g(t)}{dt}=-{k}_a\cdot {X}_g(t)\ \mathrm{when}\ \mathrm{at}\ t=0,{X}_g(0)= dose $$

$$ {V}_h\frac{d{C}_h}{dt}={Q}_h\cdotp {C}_b-\frac{Q_h\cdotp {C}_h\cdotp {R}_b}{K_{p,h}}+{k}_a\cdotp {X}_g-{CL}_{h,\mathit{\operatorname{int}}}\cdotp \frac{C_h}{K_{p,h}}\cdotp {f}_{u,p} $$

$$ {V}_1\frac{d{C}_b}{dt}=-{Q}_h\cdotp {C}_b+\frac{Q_h\cdotp {C}_h\cdotp {R}_b}{K_{p,h}}-{k}_{12}\cdotp {V}_1\cdotp {C}_b+{k}_{21}\cdotp {X}_{peripheral}-{CL}_r\cdotp {C}_b $$

$$ \frac{d{X}_{peripheral}}{dt}={k}_{12}\cdotp {V}_1\cdotp {C}_b-{k}_{21}\cdotp {X}_{peripheral} $$

where *X*_*g*_ and *X*_*peripheral*_ are the substrate amounts in the gut and peripheral compartments, *V*_*h*_ is the liver volume (1.5 L), *C*_*h*_ is the hepatic substrate concentration, *Q*_h_ is the blood flow rate of the systemic circulation to the hepatic compartment (96.6 L/h), and *C*_*b*_ is the blood substrate concentration.

**Table 2:** Physiological, experimental, and final calculated parameters for PBPK models established in this study

Parameter	Abbreviation (unit)	Duloxetine	Flunitrazepam	Quetiapine	Trazodone
Model input parameters					
Molecular weight	MW	297	313	384	372
Octanol–water partition coefficient	log*P*	4.26	1.78	2.99	3.85
Plasma unbound fraction	*f*_u,p_	0.114	0.324	0.125	0.0732
Blood–plasma concentration ratio	*R*_b_	0.843	0.921	0.852	0.805
Liver–plasma concentration ratio	*K*_p,h_	3.18	1.17	2.69	3.01
Fraction absorbed × intestinal availability	*F*_a_·*F*_g_	1	1	1	1
Absorption rate constant	*k*_a_ (1/h)	0.372 ± 0.007^a^	2.48 ± 0.05	2.86 ± 0.05	1.12 ± 0.26
Transfer rate constant	*k*_12_ (1/h)	–	0.28 ± 0.02	–	–
Transfer rate constant	*k*_21_ (1/h)	–	0.04 ± 0.01	–	–
Volume of systemic circulation	*V*_1_ (L)	755 ± 1^a^	80.7 ± 0.1	206 ± 1	66.2 ± 9.5
Hepatic intrinsic clearance	*CL*_h,int_ (L/h)	385 ± 1^a^	15.8 ± 0.1	954 ± 1	173 ± 16
Hepatic clearance	*CL*_h_ (L/h)	30.2	4.84	53.4	11.2
Renal clearance	*CL*_r_ (L/h)	3.0	0.48	5.3	1.1
Estimated values					
C_max_ in plasma	ng/mL	44.9 (0.93)^b^	9.12 (1.08)	44.2 (0.98)	491 (0.72)
AUC in plasma	ng·h/mL	1210 (1.19)	52.1 (1.02)	172 (0.95)	3610 (0.77)
Reported levels					
C_max_ in plasma	ng/mL	48.5 ± 8.3^c^	8.47^d^	45.0^e^	681 ± 128^f^
AUC in plasma	ng·h/mL	1020 ± 220	51.2	181	4670 ± 790

^a^Data are means ± standard deviations by fitting to measured concentrations. ^b^Values in parentheses are ratios to the reported/observed values. Reported/observed blood levels were taken from the literature: ^c^ [16], ^d^ [17], ^e^ [18], and ^f^ [19]

The measured plasma concentrations and the PBPK-modeled concentration profiles of the four drugs self-administered in a single oral overdose are shown in Fig. 1. The observed concentrations of duloxetine and flunitrazepam in this overdose patient were generally within the 95% CIs of the predicted plasma concentration curves.

## Discussion and conclusions

Although the observed concentrations of quetiapine and trazodone were higher than the 95% CI of the predicted plasma concentration curves, possible drug interaction effects that might have caused these observed high plasma concentrations were ruled out in this case because of the apparent wide-ranging linearity seen in overdoses in this patient and in the outputs of PBPK models (shown in Fig. 1**)** based on the recommended normal doses; quetiapine was the exception, because it exhibited unexpectedly rapid elimination in this case.

Relatively many cases of quetiapine in overdose have been reported [25]. It has been suggested that activated charcoal has an effect on the pharmacokinetics of quetiapine in overdose [26]. However, quetiapine appears to be relatively safe in overdose, presumably because of its short terminal elimination half-life [27]. In contrast, the absorption and disappearance of duloxetine were slower than those of the other three medicines experienced in this case. A low apparent permeability of duloxetine of 12.5 nm/s was determined by following the reported method in an in vitro Caco-2 monolayer system in comparison with caffeine (544 nm/s) as a reference compound [28]. Generally, gastric lavage and administration of charcoal are recommended within 1 h of overdose in clinical practice. In a case report [28], it was reported that gastric lavage could be effective when some medicine remained in the stomach. Activated charcoal reportedly prevents the absorption of controlled-release duloxetine tablets at 1 h after administration [29]. It has been reported that liposomes could potentially be effective for treating overdoses of the antidepressant amitriptyline, with reductions in the area under the concentration–time curve estimated using a PBPK model; however, the aims of that study were different from the purpose of the current study [29]. We recently proposed the practical use of PBPK models by paramedical staff in emergency clinical practice for a case of edoxaban overdose [10]. The PBPK model established in the current study predicted the time to the maximum concentration of duloxetine to be about 6 h. Therefore, even if more than 1 h has passed after administration of duloxetine, gastric lavage and the administration of activated charcoal may be effective in clinical practice.

Simplified PBPK models are useful not only in the fields of drug discovery and chemical risk assessment but also in the management of poisoning, as recently described [10]. We did not use the Michaelis-Menten equations for the in vivo intrinsic hepatic clearances in the current simplified PBPK models*.* Such models can predict plasma concentration curves, and then it can quickly be determined whether treatment with gastric lavage and activated charcoal is feasible. In this way, it may be possible to deal with individual cases by reflecting the differences in pharmacokinetics. In hospitals, a simplified PBPK model simulator could replace the need to routinely measure the blood levels of drugs. It is hoped that the results of this study based on drug monitoring data and pharmacokinetic predictions could serve as a guide when setting the treatment period in cases of overdoses of antipsychotic drugs, e.g., duloxetine and quetiapine, that are cleared differently.

## Data Availability

All data generated or analyzed during this study are included in this published article and are also available from the corresponding author on reasonable request.

## Abbreviations

CIs: Confidence intervals
PBPK: Physiologically based pharmacokinetic

## References

[1] Ereshefsky L (1996). Pharmacokinetics and drug interactions: update for new antipsychotics. J Clin Psychiatry.

[2] Zhou SF (2009). Polymorphism of human cytochrome P450 2D6 and its clinical significance: part II. Clin Pharmacokinet.

[3] Menchetti M, Gozzi BF, Saracino MA, Mercolini L, Petio C, Raggi MA (2009). Non-fatal overdose of duloxetine in combination with other antidepressants and benzodiazepines. World J Biol Psychiatry.

[4] Paulzen M, Hiemke C, Grunder G (2009). Plasma levels and cerebrospinal fluid penetration by duloxetine in a patient with a non-fatal overdose during a suicide attempt. Int J Neuropsychopharmacol.

[5] Kruithof MK, Bruins NA, van Roon EN (2011). Coma after overdose with duloxetine. Ann Pharmacother.

[6] Pellicciari A, Balzarro B, Scaramelli A, Porcelli S, Serretti A, De Ronchi D (2012). Generalized tonic-clonic seizure secondary to duloxetine poisoning: a short report with favorable outcome. Neurotoxicology.

[7] Scanlon KA, Stoppacher R, Blum LM, Starkey SJ (2016). Comprehensive duloxetine analysis in a fatal overdose. J Anal Toxicol.

[8] Alibegovic A, Kariz S, Volavsek M (2019). Fatal overdose with a combination of SNRIs venlafaxine and duloxetine. Forensic Sci Med Pathol.

[9] Yamazaki-Nishioka M, Kogiku M, Noda M, Endo S, Takekawa M, Kishi H (2019). Pharmacokinetics of anticoagulants apixaban, dabigatran, edoxaban and rivaroxaban in elderly Japanese patients with atrial fibrillation treated in one general hospital. Xenobiotica.

[10] Adachi K, Tuchiya J, Beppu S, Nishiyama K, Shimizu M, Yamazaki H (2020). Pharmacokinetics of anticoagulant edoxaban in overdose in a Japanese patient transported to hospital. J Pharm Health Care Sci.

[11] Chen X, Liang C, Cui L, Le J, Qian Z, Zhang R (2019). A rapid LC-MS/MS method for simultaneous determination of quetiapine and duloxetine in rat plasma and its application to pharmacokinetic interaction study. J Food Drug Anal.

[12] Patel BN, Sharma N, Sanyal M, Shrivastav PS (2008). High throughput and sensitive determination of trazodone and its primary metabolite, m-chlorophenylpiperazine, in human plasma by liquid chromatography-tandem mass spectrometry. J Chromatogr B Analyt Technol Biomed Life Sci.

[13] Furugen A, Nishimura A, Kobayashi M, Umazume T, Narumi K, Iseki K (2019). Quantification of eight benzodiazepines in human breastmilk and plasma by liquid-liquid extraction and liquid-chromatography tandem mass spectrometry: application to evaluation of alprazolam transfer into breastmilk. J Pharm Biomed Anal.

[14] Zhao RK, Cheng G, Tang J, Song J, Peng WX (2009). Pharmacokinetics of duloxetine hydrochloride enteric-coated tablets in healthy Chinese volunteers: a randomized, open-label, single- and multiple-dose study. Clin Ther.

[15] Koller D, Zubiaur P, Saiz-Rodriguez M, Abad-Santos F, Wojnicz A (2019). Simultaneous determination of six antipsychotics, two of their metabolites and caffeine in human plasma by LC-MS/MS using a phospholipid-removal microelution-solid phase extraction method for sample preparation. Talanta..

[16] Gajula R, Maddela R, Babu Ravi V, Inamadugu JK, Pilli NR (2013). A rapid and sensitive liquid chromatography-tandem mass spectrometric assay for duloxetine in human plasma: its pharmacokinetic application. J Pharm Anal.

[17] Gafni I, Busto UE, Tyndale RF, Kaplan HL, Sellers EM (2003). The role of cytochrome P450 2C19 activity in flunitrazepam metabolism in vivo. J Clin Psychopharmacol.

[18] Grimm SW, Richtand NM, Winter HR, Stams KR, Reele SB (2006). Effects of cytochrome P450 3A modulators ketoconazole and carbamazepine on quetiapine pharmacokinetics. Br J Clin Pharmacol.

[19] Farkas D, Volak LP, Harmatz JS, von Moltke LL, Court MH, Greenblatt DJ (2009). Short-term clarithromycin administration impairs clearance and enhances pharmacodynamic effects of trazodone but not of zolpidem. Clin Pharmacol Ther.

[20] Kamiya Y, Otsuka S, Miura T, Takaku H, Yamada R, Nakazato M (2019). Plasma and hepatic concentrations of chemicals after virtual oral administrations extrapolated using rat plasma data and simple physiologically based pharmacokinetic models. Chem Res Toxicol.

[21] Adachi K, Suemizu H, Murayama N, Shimizu M, Yamazaki H (2015). Human biofluid concentrations of mono (2-ethylhexyl) phthalate extrapolated from pharmacokinetics in chimeric mice with humanized liver administered with di(2-ethylhexyl) phthalate and physiologically based pharmacokinetic modeling. Environ Toxicol Pharmacol.

[22] Miyaguchi T, Suemizu H, Shimizu M, Shida S, Nishiyama S, Takano R (2015). Human urine and plasma concentrations of bisphenol a extrapolated from pharmacokinetics established in in vivo experiments with chimeric mice with humanized liver and semi-physiological pharmacokinetic modeling. Regul Toxicol Pharmacol.

[23] Uchimura T, Kato M, Saito T, Kinoshita H (2010). Prediction of human blood-to-plasma drug concentration ratio. Biopharm Drug Dispos.

[24] Takano R, Murayama N, Horiuchi K, Kitajima M, Kumamoto M, Shono F (2010). Blood concentrations of acrylonitrile in humans after oral administration extrapolated from in vivo rat pharmacokinetics, in vitro human metabolism, and physiologically based pharmacokinetic modeling. Regul Toxicol Pharmacol.

[25] Montgomery SA (2008). Tolerability of serotonin norepinephrine reuptake inhibitor antidepressants. CNS Spectr.

[26] Isbister GK, Friberg LE, Hackett LP, Duffull SB (2007). Pharmacokinetics of quetiapine in overdose and the effect of activated charcoal. Clin Pharmacol Ther.

[27] Pollak PT, Zbuk K (2000). Quetiapine fumarate overdose: clinical and pharmacokinetic lessons from extreme conditions. Clin Pharmacol Ther.

[28] Kamiya Y, Takaku H, Yamada R, Akase C, Abe Y, Sekiguchi Y (2020). Determination and prediction of permeability across intestinal epithelial cell monolayer of a diverse range of industrial chemicals/drugs for estimation of oral absorption as a putative marker of hepatotoxicity. Toxicol Rep.

[29] Howell BA, Chauhan A (2010). A physiologically based pharmacokinetic (PBPK) model for predicting the efficacy of drug overdose treatment with liposomes in man. J Pharm Sci.

